# Modulation of collagen-induced arthritis by adenovirus-mediated intra-articular expression of modified collagen type II

**DOI:** 10.1186/ar3074

**Published:** 2010-07-08

**Authors:** Bo Tang, David L Cullins, Jing Zhou, Janice A Zawaski, Hyelee Park, David D Brand, Karen A Hasty, M Waleed Gaber, John M Stuart, Andrew H Kang, Linda K Myers

**Affiliations:** 1 Department of Medicine, University of Tennessee Health Science Center, 956 Court Avenue, Memphis, Tennessee 38163, USA; 2 Department of Biomedical Engineering, University of Tennessee Health Science Center, 920 Madison, Suite 407, Memphis, Tennessee 38163 USA; 3 Department of Orthopedics, University of Tennessee Health Science Center, 1211 Union Avenue, Suite 520, Memphis, Tennessee 38104 USA; 4 Research Service, Veterans Affairs Medical Center, 1030 Jefferson Avenue, Memphis TN 38104 USA; 5 Department of Pediatrics, University of Tennessee Health Science Center, 50 North Dunlap, Room 401, Memphis TN 38163 USA

## Abstract

**Introduction:**

Rheumatoid arthritis (RA) is a systemic disease manifested by chronic inflammation in multiple articular joints, including the knees and small joints of the hands and feet. We have developed a unique modification to a clinically accepted method for delivering therapies directly to the synovium. Our therapy is based on our previous discovery of an analog peptide (A9) with amino acid substitutions made at positions 260 (I to A), 261 (A to B), and 263 (F to N) that could profoundly suppress immunity to type II collagen (CII) and arthritis in the collagen-induced arthritis model (CIA).

**Methods:**

We engineered an adenoviral vector to contain the CB11 portion of recombinant type II collagen and used PCR to introduce point mutations at three sites within (CII_124-402, 260A, 261B, 263D_), (rCB11-A9) so that the resulting molecule contained the A9 sequence at the exact site of the wild-type sequence.

**Results:**

We used this construct to target intra-articular tissues of mice and utilized the collagen-induced arthritis model to show that this treatment strategy provided a sustained, local therapy for individual arthritic joints, effective whether given to prevent arthritis or as a treatment. We also developed a novel system for *in vivo *bioimaging, using the firefly luciferase reporter gene to allow serial bioluminescence imaging to show that luciferase can be detected as late as 18 days post injection into the joint.

**Conclusions:**

Our therapy is unique in that we target synovial cells to ultimately shut down T cell-mediated inflammation. Its effectiveness is based on its ability to transform potential inflammatory T cells and/or bystander T cells into therapeutic (regulatory-like) T cells which secrete interleukin (IL)-4. We believe this approach has potential to effectively suppress RA with minimal side effects.

## Introduction

Rheumatoid arthritis (RA) is a systemic disease with polyarticular manifestation of chronic inflammation in multiple articular joints, including the knees and small joints of the hands and feet. The current systemic anti-TNF-α therapies ameliorate disease in 60% to 70% of RA patients [1]. However, biologics must be given systemically in relatively high dosages to achieve constant therapeutic levels in the joints, and significant side effects have been reported [2].

Gene therapy may provide an effective alternative to drug delivery for the treatment of arthritis [3]. Although various strategies have been tested, those that target gene delivery to the synovial lining of joints have made the most experimental progress [3,4]. This strategy has shown efficacy in several experimental models of RA [5-7]. For this reason, we have developed a unique modification to a clinically acceptable method of gene delivery to allow delivery of the gene product directly to the synovium. Our therapy is based on our previous discovery of an analog peptide (A9) of type II collagen (CII) with amino acid substitutions made at positions 260 (I to A), 261 (A to B), and 263 (F to N) that could profoundly suppress immunity to CII and arthritis in the collagen-induced arthritis (CIA) model [8]. Such collagen peptides containing specially designed substitutions and expressed as a gene products may provide an ideal choice to be delivered to the joints.

We engineered an adenoviral gene-based therapy and showed that this treatment strategy provided a sustained, local therapy for individual arthritic joints. Our therapy is unique in that we target synovial cells to ultimately shut down T cell-mediated inflammation. Its effectiveness is based on its ability to transform potential inflammatory T cells and/or bystander T cells into therapeutic (regulatory-like) T cells [8]. They are potentially safer than current therapies because they contain a modification of an endogenous naturally occurring protein, used to interrupt the autoimmune T cell attack and allow for tissue repair. We believe this approach has the potential to become applicable for treatment of RA.

## Materials and methods

### Preparation of tissue-derived type II collagen

Native CII was solubilized from fetal calf articular cartilage by limited pepsin-digestion and purified as described earlier [9]. The purified collagen was dissolved in cold 0.01 M acetic acid at 4 mg/ml and stored frozen at -70°C until used.

### Animals

DBA/1 mice were obtained from the Jackson Laboratories and raised in our animal facility. They were fed standard rodent chow (Ralston Purina Co., St. Louis, MO, USA) and water *ad libitum*. The environment was specific pathogen-free and sentinel mice were tested routinely for mouse hepatitis and Sendai viruses. All animals were kept until the age of 7 to 10 weeks before being used for experiments, which were conducted in accordance with approved Institutional Animal Care and Use Committee (IACUC) protocols.

### Immunization

CII was solubilized in 0.01 M acetic acid at a concentration of 4 mg/ml and emulsified with an equal volume of complete Freund's adjuvant (CFA) containing 4 mg/ml of *Mycobacterium tuberculosis *strain H37Ra (Difco Microbiology Products, Becton Dickinson, NJ, USA) [10]. Each mouse received 100 μg of CII emulsified in CFA intradermally at the base of the tail.

### Generation of replication-defective, recombinant adenoviral vector expressing modified CB11

Recombinant adenovirus carrying cDNA for rCB11-A9 was generated using a BD Adeno-X Expression System (BD Biosciences Clontech (San Jose, California, USA)), which incorporates the rCB11-A9 expression cassette into a replication-incompetent (ΔE1/ΔE3) human adenoviral type 5 (Ad5) genome. All work was conducted in accordance with approved Institutional Biosafety Committee (IBC) protocols. In brief, an 834 bp of full-length murine CB11 gene was PCR-amplified from murine spleen cDNA and cloned into the PCR2 vector (Invitrogen, Carlsbad, California, USA). We introduced three point mutations (I260A, A261B, and F263N) within the immunodominant T cell determinant of CB11 (CII_124-402_) to generate an rCB11-A9 construct. The rCB11-A9 cDNA was then excised with BamHI/EcoR I and subcloned into the same sites of the pShuttle2 vector to construct an rCB11-A9 specific expression cassette. For *in vivo *bioimaging analysis, a cDNA encoding the luciferase gene was also subcloned into the pShuttle2 to establish the Adeno-X-luciferase expression cassette. To produce recombinant adenoviral DNA containing rCB11-A9 or luciferase, we excised the expression cassettes from recombinant pShuttle2 plasmid DNA by digesting with I-Ceu I and PI-Sce I and ligated the expression cassettes with prelinearized BD Adeno-X Viral DNA (I-Ceu I and PI-Sce I digested). Low passage HEK293 cells were transfected with the resultant recombinant adenoviral DNA using the calcium phosphate method [11]. The recombinant adenoviral particles were harvested by lysing transfected cells. The resultant AdenoX-rCB11-A9 is a replication-incompetent recombinant adenovirus. High titer viral stocks (about 10^8 ^to 10^9 ^plaque forming units (pfu)/ml) were obtained by amplifying recombinant adenovirus in HEK 293 cells. A construct (pShuttle2-lacZ) was included in the BD Adeno-X Expression System and recombinant AdenoX-lacZ was generated as described above and used as a control. The recombinant adenoviral titers were determined by BD Adeno-X Rapid Titer Kit [11,12].

### Production and purification of recombinant CB11 and CB11-A9

In some experiments, a baculoviral expression system was used to produce rCB11 (CII_124-402_^bac^) in insect cells essentially as described earlier [13]. The cDNA for both recombinant CB11 and CB11-A9 (rCB11 and rCB11-A9) were subcloned into a Gateway entry vector (Invitrogen, Carlsbad, California, USA) and validated. The resultant Gateway entry vectors containing either rCB11 or rCB11-A9 were ligated with BaculoDirect Linear DNA (Invitrogen, Carlsbad, California, USA) and transfected into Sf9 insect cells. Supernatants from lysed insect cells were collected and screened for expression by performing SDS-PAGE and western blot analysis. After validated, high titers of recombinant baculovirus were obtained by re-infecting Sf9 cells twice and supernatants collected from lysed cells. To express the recombinant proteins Hi5 cells was infected with high titer of baculovirus. Supernatants from cultured Hi5 cells were harvested by centrifugation and the recombinant proteins purified by gel filtration and cation exchange chromatography, and dialyzed in dilute acetic acid.

### Synovial injections

The hind ankle joints of DBA/1 mice were injected intra-articularly with 10 ul of adenoviral vector 1 × 10^7 ^pfu of adenovirus, containing the DNA for either luciferase, rCB11-A9, or Lac-Z. In some experiments, selected mice were injected intraperitoneally with luciferin, and the expression of the transgene (luciferase) was detected by bioluminescent imaging using a liquid nitrogen cooled CCD camera (Photometric Chemipro, Roper Scientific, NJ, USA) mounted on a dark box one hour later. Images were acquired and analyzed using Metamorph software (Universal Imaging Co., Dowlington, PA, USA).

### Measurement of the incidence and severity of arthritis

The incidence and severity of arthritis were determined by visually examining each forepaw and hindpaw and scoring them on a scale of 0 to 4 as described previously [10]. Scoring was conducted by two examiners, one of whom was unaware of the identity of the treatment groups. Each mouse was scored thrice weekly beginning three weeks post immunization and continuing for eight weeks. The incidence of arthritis (number of animals with one or more arthritic limbs) and mean severity score (sum of the severity scores/total number of animals in the group) was recorded at each time point.

In a prevention protocol, four groups of 10 DBA/1 mice each were administered intra-articularly in the ankles, either adenoX-rCB11-A9 or adenoX-LacZ. The mice were immunized with CII/CFA either three or seven days after the injection.

In a treatment protocol, groups of three DBA/1 mice were immunized with CII/CFA and at the time arthritis reached a severity score of two or greater, the mice were administered intra-articularly in the hind ankles either adenoX-rCB11-A9 or adenoX-LacZ.

### Measurement of serum antibody titers

Mice were bled at six weeks after immunization and sera were analyzed for antibodies reactive with native CII using a modification of an ELISA previously described [10]. Serial dilutions of a standard serum were added to each plate. From these values, a standard curve was derived by computer analysis using a four-parameter logistic curve. Results are reported as units of activity, derived by comparison of test sera with the curve derived from the standard serum which was arbitrarily defined as having 50 units of activity. Reactivity to CII was not detected in sera obtained from normal mice.

### Measurement of cytokines

Groups of three DBA/1 mice were administered intra-articularly either adenoX-rCB11-A9 or adenoX-LacZ and the mice were immunized with CII/CFA three days after the injection. Draining lymph node cells were harvested 14 days after the immunization and cultured (5 × 10^6 ^cells/ml) with 100 μg/ml of either the mouse collagen immunodominant peptide, Ova (negative control), or purified protein derivative (PPD) (positive control). Supernatants were collected 72 hours later and analyzed for the presence of multiple cytokines (IL-4, IL-5, IL-10, IL-2, interferon (IFN)γ, and IL-17 by a Bio-plex mouse cytokine assay (Bio-Rad, Hercules, CA, USA) according to the manufacturer's protocol. Values are expressed as picograms per ml and represent the mean values for each group.

### Statistical analysis

The incidence of arthritis in various groups of mice was compared using Fisher's Exact Test. Mean severity scores and antibody levels were compared using Student's t test.

## Results

### An adenoviral construct efficiently transfers an exogene into arthritic synovial tissues in collagen-induced arthritis

**Figure 1:**
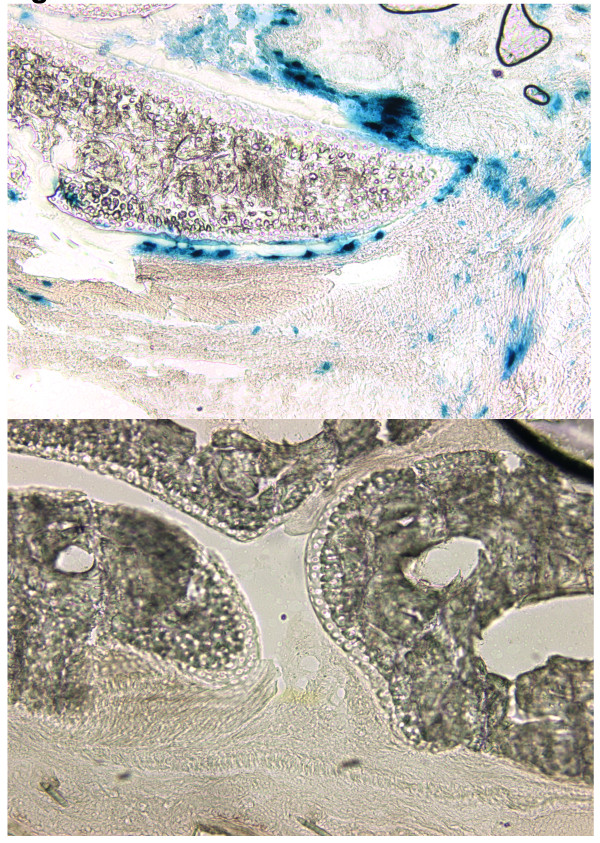
**Localization of adenovirus-expressed recombinant protein in arthritic mouse paws**. Two DBA/1 mice were immunized with type II collagen/complete Freund's adjuvant and one week later injected intra-articularly (into hind ankles) with 10 μl containing 10^× 7 ^total plaque forming units adenoviral particles encoding Lac-Z. The animals were sacrificed 48 hours later and the tissues photographed using a reverse phase microscope (50×). In the upper panel, the tissues were incubated with beta galactosidase substrate. The majority of the cells containing Lac-Z (upper panel, stained blue) appear to be fibroblast-like synoviocytes lining the surface of the synovium, although staining can also be detected in monocyte-like synoviocytes. The uninjected hindpaws were used as controls for each animal (lower panel). The data shown are representative of data obtained by analyzing multiple sections of each hindpaw.

Using a replication-defective, recombinant adenovirus, we incorporated the cDNA for lac-Z, and assessed the transfection efficiency of the recombinant adenovirus delivered into arthritic ankles of DBA/1 mice previously immunized with CII/CFA. Each hind ankle was injected intra-articularly with 10^7 ^pfu of the adenoviral particles. Forty-eight hours later, the animals were sacrificed and histology was performed on the involved joints. As shown in Figure 1, staining with β-galactosidase clearly demonstrated that the adenovirus-expressed gene product was present in synovial cells, lining the surface of the synovium near the cartilage surface (Figure 1). Although most of the transfected cells are fibroblast-like synoviocytes, a smaller number of monocyte-like synovial cells were also transfected. These data confirm that arthritic synovial cells can be readily transfected with adenoviral constructs and that an adenovirus carrying gene can be efficiently expressed.

### Development of the baculovirus construct for modified collagen (rCB11-A9) expression and evaluation of its immunogenicity

To develop a unique collagen-based therapy, we built upon our previous work demonstrating that a synthetic peptide of CII, which contained three amino acid substitutions (A9), could effectively suppress arthritis. We used PCR to introduce three point mutations within the CB11 portion of recombinant type II collagen (CII_124-402,260A, 261B, 263D_), (rCB11-A9) so that the resulting molecule contained the A9 sequence at the exact site of the wild-type sequence. To test for safety, we developed a baculovirus construct and expressed the rCB11-A9 protein in drosophila cells, because insect cells express a modest activation of lysine hydroxylase and hydroxylysine glycosyltransferase, allowing partial glycosylation of the product. This system closely mimics the post-translational system of mammalian cells. The baculovirus-expressed collagen was purified, emulsified with CFA, and used to immunize DBA/1 mice to observe for the development of arthritis. We found that rCB11-A9 was unable to induce either arthritis or antibodies to CII (Table 1). On the other hand, the unmodified control rCB11-induced arthritis at its expected incidence of 40% as well as inducing a significant antibody response to murine CII (Table 1). These data suggest that rCB11-A9 will be safer than many conventional therapies, if used to treat arthritis because it is non-immunogenic and non-arthritogenic.

**Table 1:** A9-modified recombinant CB11 is not arthritogenic

Format of collagenous immunogen^a^	Incidence^b^	Antibodies to CII^c^
rCB11-A9/CFA	0/5 (0%)	2.5 ± 1, *P *< 0.05
rCB11/CFA	2/5 (40%)	21.3 ± 5

a. The baculovirus-expressed products, both rCB11 (recombinant CII_124-402)_) and rCB11-A9 (recombinant CII_124-402,260A, 261B, 263D_) were collected, emulsified with complete Freund's adjuvant (CFA), and used to immunize groups of five DBA/1 mice to observe for the development of arthritis. We found that modified rCB11-A9 was unable to induce either arthritis or significant antibody titers to type II collagen (CII) while the control unmodified rCB11-induced arthritis at its expected incidence of 40% as well as inducing a greater antibody response to murine CII.

b. Incidence is reported at six weeks following immunization.

c. Antibody levels were measured by ELISA and reported as arbitrary units based on comparison to a standard antiserum run simultaneously.

### Development of a method for *in vivo *bioimaging to track the duration of gene expression

**Figure 2:**
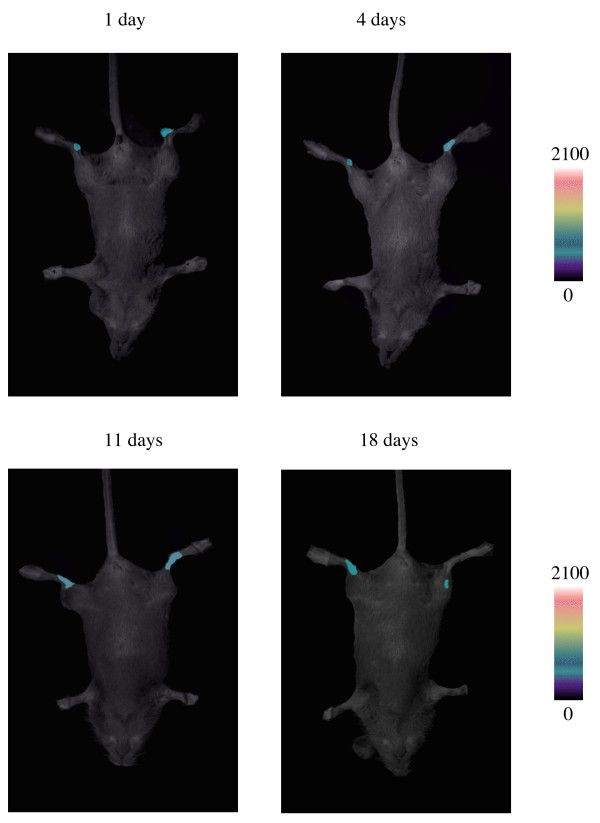
**Adenoviral-mediated gene transfer in joints of live mice**. Two mice were injected with 10 μl of adenoviral particles (1 × 10^7 ^plaque forming units) expressing luciferase into each of the hind ankle joints. At various time points, the mice were injected intraperitoneally with luciferin, and expression of the transgene (luciferase) detected by bioluminescent imaging at one hour after administration of luciferin. The injected joints clearly showed the expression of luciferase, as indicated by the green luminescent color and the expression of luciferase could be detected as late as 18 days post injection.

Noninvasive bioimaging is an exciting new development that can be applied to clinical diseases to monitor the duration of gene expression and determine the extent of the therapeutic effect. As adenoviral constructs typically can be transcribed but not replicated with cell division, it was important to predict the length of time the introduced therapy might be effective. We developed a system for *in vivo *bioimaging in which the firefly luciferase reporter gene was incorporated into our adenoviral vector and this construct was injected into murine ankle joints. Serial bioluminescence imaging of gene expression was performed on days 1, 3, 12 and 18 following intraperitoneal injection of luciferin, the substrate of luciferase. As shown in Figure 2, the injected sites of joints clearly showed the expression of luciferase, as indicated by the green luminescent color and the expression of luciferase could be detected as late as 18 days post injection. By day 21 the green color was no longer detectable. Taken together, these data confirm that a transgene carried by the adenoviral vector gene can be efficiently transferred into the joints and a sustained release of expression can be successfully achieved.

### Evaluation of the potency of the AdenoX-rCB11-A9 construct in suppression of CIA

**Figure 3:**
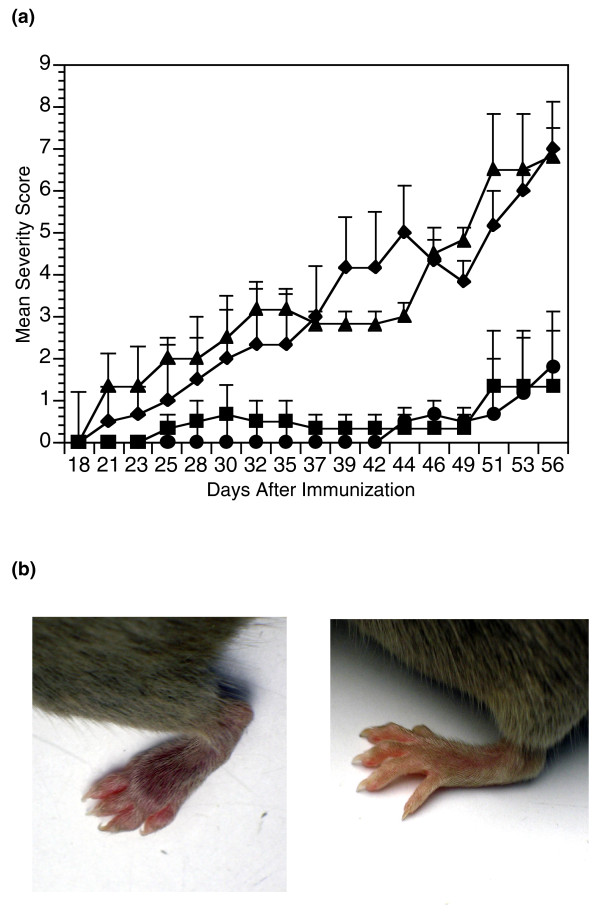
**Treatment with adenoX-rCB11-A9 can prevent CIA**. **(a) **Groups of 10 DBA/1 mice were administered intra-articularly either adenoX-rCB11-A9 (square, circle) or adenoX-LacZ (triangle, diamond). The mice were immunized with type II collagen/complete Freund's adjuvant either three (square, triangle) or seven (circle, diamond) days after the injection and all mice were observed for the development of arthritis. The data points reflect the mean severity score (sum of the severity scores/total number of animals in the group) at each time point. As shown the adenoX-rCB11-A9 was extremely effective at preventing the development of arthritis, whether the mice were immunized three days prior to immunization (final severity scores 1.3 ± 1.5 vs 6.8 ± 5.3, *P *≤ 0.025) or seven days prior to immunization (final severity scores 1.8 ± 2.0 vs 7.0 ± 2.5, *P *≤ 0.005). The final incidence of arthritis was (square = 20%, triangle = 90%, *P *≤ 0.003) and (circle = 20%, diamond= 100%, *P *≤ 0.0004). **(b) **Photographs of an arthritic hind paw from a DBA/1 mouse injected with adenoX-lacZ (left panel) and a hind paw from a DBA/1 mouse injected with adenoX-rCB11-A9 (right panel).

All the previous data suggest that local expression of rCB11-A9 in arthritic joints will be able to effectively modulate CIA. To test this hypothesis the rCB11-A9 was incorporated into the adenoviral genome and the resulting construct (AdenoX-rCB11A9) tested. To evaluate potency in the treatment of arthritis, DBA/1 mice were injected intra-articularly (in the hind ankles) with the adenoX-rCB11-A9 either three or seven days prior to immunization with CII/CFA and were observed for the development of arthritis. Control mice were injected with adenoX-lacZ. As predicted, the mice treated with the adenoX-rCB11-A9 demonstrated a significant decrease in the severity of arthritis as manifested by the severity scores and visual inspection (Figure 3, panels a and b). The control adenoX-Lac-Z construct had no effect. Concordant with a decrease in the incidence and severity of arthritis, antibody production to CII was significantly decreased (Table 2). The hindpaws injected with AdenoX-rCB11A9 were profoundly affected when compared with adenoX-lacZ injected control hindpaws, (severity scores of 0 vs 2.8 ± 2.7, *P *≤ 0.025 if injected three days prior to immunization and 0 vs 2.2 ± 1.8, *P *≤ 0.01 if injected seven days prior to immunization). The non-injected forepaws developed arthritis with attenuated severity (severity scores of 1.3 ± 1.5 vs 4.8 ± 2.1, *P *≤ 0.01 when treated three days prior, to imunization or 1.8 ± 2 vs 4.8 ± 1.5, *P *≤ 0.01 when treated seven days prior to immunization). These data indicate that therapy with adenoX-rCB11-A9 significantly down regulated the immune responses to CII *in vivo *and attenuated the development of arthritis.

**Table 2:** AdenoX-rCB11-A9 treatment suppresses anti-CII antibodies

Antibodies to CII in treated mice	
**Treatment^a^**	**Antibodies to CII^b^**

AdenoX-rCB11-A9 (imm CII/CFA 3 days later)	19.6 ± 2, *P *< 0.05
AdenoX-rCB11-A9 (imm CII/CFA 7 days later)	16.2 ± 2 *P *< 0.005

AdenoX-lacZ (imm CII/CFA 3 days later)	37.2 ± 10
AdenoX-lacZ (imm CII/CFA 7 days later)	45.0 ± 9

a. Four groups of 10 DBA/1 mice each were administered intra-articularly either adenoX-rCB11-A9 (adenoviral construct with DNA encoding (CII_124-402,260A, 261B, 263D_) or adenoX-LacZ (adenoviral construct with DNA encoding LacZ). The mice were immunized with type II collagen/complete Freund's adjuvant (CII/CFA) either three or seven days after the injection and sera was collected six weeks after the immunization to test for antibodies to CII. As noted the adenoX-rCB11-A9 was extremely effective at suppressing the development of antibodies to CII, irregardless of whether the mice were immunized three days or seven days later.

b. Antibody levels were measured by ELISA and reported as arbitrary units based on comparison to a standard antiserum run simultaneously.

### Mechanism of suppression

We have reported that a major component of the mechanism of action for the synthetic peptide analog A9 is its ability to cause T cells to secrete a suppressive cytokine profile. Its effectiveness is based on its ability to transform potential inflammatory T cells and/or bystander T cells into therapeutic (regulatory-like) T cells [8,14]. Based on our previous observation that the activated antigen-specific T cells found in draining lymph nodes can accurately reflect the T cell responses of arthritic joints [15], we examined the secretion of a panel of cytokines IFN-γ and IL-2 (Th1), IL-10 and IL-4 (Th2) and IL-17 (Th17), by testing supernatants from draining lymph node cells of mice cultured with the murine immunodominant determinant. We found that following treatment with adenoX-CB11-A9, the Th1 and Th17 cytokine responses to murine CII were significantly decreased compared with those induced following treatment with adenoX-lacZ. Similarly, the Th2 cytokines IL-5 and IL-10 were decreased. On the other hand, treatment with the adenoX-rCB11-A9 induced a significantly greater IL-4 response to murine CII when compared with the lac-Z control (Table 3). These data are consistent with the concept that IL-4 has a unique role in the suppression of arthritis that is only partially duplicated by other Th2-type cytokines in the absence of IL-4 [16]. Taken together these data suggest that the adenoX-rCB11-A9 therapy may work by inducing a population of T cells to redirect their cytokine response to secrete predominantly IL-4, a cytokine known to ameliorate arthritis. The ability to induce a population of regulatory-like T cells to secrete suppressive cytokines in the presence of murine CII as well as the ability to redirect inflammatory cells toward a more suppressive phenotype may explain the profound downregulatory effects adenoX-rCB11-A9 has on CIA.

**Table 3:** Cytokine responses in mice treated with gene therapy

		**Cytokines(pg/ml)**					
		
Treatment	Antigen	IL-2	IFN-γ	IL-17	IL-4	IL-5	IL-10
AdenoX-Lac-z	Ova	131 ± 12	137 ± 14	280 ± 22	1 ± 1	82 ± 10	18 ± 8
AdenoX-Lac-z	Collagen peptide	1,405 ± 120	643 ± 25	6,364 ± 220	7 ± 3	762 ± 44	214 ± 17
AdenoX-Lac-z	PPD	631 ± 50	2,451 ± 50	8,612 ± 267	3 ± 2	820 ± 63	262 ± 21

AdenoX-rCB11A9	Ova	120 ± 15	164 ± 22	250 ± 25	2 ± 1	75 ± 8	16 ± 9
AdenoX-rCB11A9	Collagen peptide	152 ± 14	143 ± 15	286 ± 25	44 ± 6	203 ± 21	47 ± 11
AdenoX rCB11A9	PPD	776 ± 62	2,620 ± 232	8,633 ± 547	2 ± 2	778 ± 83	245 ± 26

Groups of three DBA/1 mice were administered intra-articularly either adenoX-rCB11-A9 or adenoX-LacZ and the mice were immunized with type II collagen/complete Freund's adjuvant (CII/CFA) three days after the injection. Draining lymph node cells were harvested 14 days after the immunization and cultured (5 × 10^6 ^cells/ml) with 100 μg/ml of the indicated antigens, either Ova (negative control), the mouse collagen immunodominant wild type peptide, or Purified Protein Derivative (PPD) (positive control). Supernatants were collected 72 hours later and analyzed for the presence of the indicated cytokines. Values are expressed as picograms per ml and represent the mean values for each group.

Cytokines IL-2, IFN-γ, IL-17, IL-5, and IL-10 were all significantly higher in response to the mouse collagen immunodominant peptide in the adenoX-lacZ treated mice compared with mice treated with adenoX-rCB11-A9 (*P *≤ 0.05). On the other hand, IL-4 was significantly greater in the mice treated with aden-rCB11-A9 (*P *≤ 0.05).

### Potency of AdenoX-rCB11-A9 in suppression of CIA when injected after the onset of arthritis

**Figure 4:**
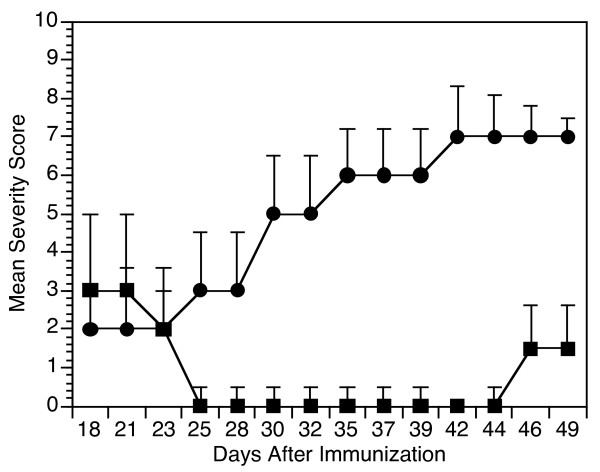
**Treatment with adenoX-rCB11-A9 can suppress collagen-induced arthritis when administered after the development of established arthritis**. Groups of 3 DBA/1 mice were immunized with type II collagen/complete Freund's adjuvant and at the time arthritis reached a severity score of two or greater, the mice were administered intra-articularly either adenoX-rCB11-A9 (square) or adenoX-LacZ (circle). All mice were observed for the development of arthritis. The data points reflect the mean severity score (sum of the severity scores/total number of animals in the group) at each time point. As noted the adenoX-rCB11-A9 was extremely effective at treating established arthritis, causing a reversal of the severity of the disease which persisted for a full two weeks (severity scores of 0 vs 5.1 ± 2.1, *P *< 0.05 on day 30 after immunization and 0 vs 7.1 ± 2.2; *P *≤ 0.05 on day 44).

In clinical situations, gene therapy is more likely to be used therapeutically rather than to prevent disease. To determine the effectiveness of this treatment on well-established arthritis, we immunized mice with CII/CFA and at the onset of arthritis (severity score greater than two), we introduced the adenoX-rCB11-A9 into the joints. As shown in Figure 3, the mice injected with the adenoX-rCB11-A9 had a reversal of arthritis, reaching severity scores of 0 within five days and the modulation of arthritis lasted approximately three weeks after the injection. Control mice injected with the adenoX-lacZ control did not improve and progressed to develop a more severe arthritis (Figure 4). The time course for the modulation of arthritis fits quite accurately with the time course predicted by the bioimaging data.

## Discussion

Our aim was to engineer an adenoviral-based therapy designed to make synovial cells secrete a modified naturally produced molecule, type II collagen, thereby providing a sustained, local therapy for individual arthritic joints. This approach is attractive because joints are discrete, accessible cavities that can be readily injected. Many different genes have been evaluated for their ability to treat animal models of RA [17]. These have led to several clinical trials, confirming the feasibility and in a preliminary fashion, safety of gene transfer to human arthritic joints [3,4,18,19].

Recently, several studies using adenoviral-mediated gene transfer of therapeutic genes for animal model treatment have been reported [7,20-22]. Adenoviruses carry their genetic material in the form of double-stranded DNA. When these viruses infect a host cell, the DNA molecule is left free in the nucleus of the host cell, and is transcribed, but not replicated. The advantages of this therapy are two-fold [23]. The treatment gives a sustained release of the material directly into the joint cavity, greatly decreasing the amount of material required and the number of injections necessary. Second, the absence of integration into the host cell's genome lessens the possibility of permanent side effects, and prevents the possibility of malignant-type transformations. Although concerns about the safety of adenovirus vectors have been raised, newer genetically crippled versions of the virus together with modified or deleted capsid sequences have demonstrated an increased safety and potential for stable transgene expression [23].

Most gene transfer strategies for treatment of RA are currently broad based, designed for introducing cytokines [3,4,18-22]. The capability of collagen peptides to act locally to induce T cells to secrete suppressive cytokines in a limited environment makes them interesting as potential therapeutic reagents in suppressing RA. Our therapy is based on our previous discovery of an analog peptide (A9) with amino acid substitutions made at positions 260 (I to A), 261 (A to B), and 263 (F to N) that profoundly suppressed immunity to CII and arthritis. In a mouse model of RA, A9 protein therapy achieved a dramatic arrest in the overall disease progression as judged by clinical, histopathological, and immunological manifestations of arthritis [8]. We now demonstrate *in vivo *immunomodulatory properties of rCB11-A9, supporting its therapeutic potential in the treatment of inflammatory autoimmune disorders. Such collagen peptides containing specially designed substitutions and expressed as gene products may provide an ideal choice to be delivered to the joints. The advantages over conventional therapies include the ease with which they can be injected at the site of the inflammation, targeting the specific arthritogenic lymphocytes that initiate and perpetuate joint inflammation, and transforming potential inflammatory T cells and/or bystander T cells into therapeutic (regulatory-like) T cells. Our results suggest that the effects are primarily localized to the joints, although we have not performed biodistribution studies. They are potentially safer than current therapies because they contain a modification of an endogenous naturally occurring protein. The use of the gene therapy overcomes the problems of rapid degradation and short half-life of small synthetic proteins *in vivo*.

Another great advantage of gene delivery to the synovial cells is that they contain the enzymatic apparatus to apply post-translational modifications, including the hydroxylation and glycosylation of lysine residues, which occur in chondrocyte synthesized CII, but not synthetic peptides. It is known that CII peptide fragments derived from the cyanogen bromide digestion of native CII are immunologically more active than chemically synthesized peptides [24,25]. It is now generally accepted that part of the T cell response to cartilage-derived CII is dependent upon the presence of glycosylated determinants, which stabilize major histocompatibility complex/T cell receptor (MHC/TCR) interaction or act as part of the epitope [24-27].

Despite these advantages, it should be noted that there is no consensus concerning the ideal vector for human gene therapies. For example, patients can carry pre-existing neutralizing antibodies to adenoviral vectors or develop them after the first injections, reducing their effectiveness. Although scientific breakthroughs continue to move gene therapy toward mainstream medicine, future research should enhance clinical applications of a collagen-based gene therapy for RA.

## Conclusions

In summary, our studies demonstrate that: recombinant CB11-A9 adenovirus can efficiently transfer and express exogenes in joints and synovial tissue; the expression persists for at least 18 days after the injection; and this type of therapy is effective at both prevention and treatment of autoimmune arthritis. These data strongly support our hypothesis that adenoviral-mediated modified collagen-type therapies can suppress arthritis and transform activated T cells and bystander T cells into therapeutic (regulatory-like) T cells. Gene therapy has emerged as an effective and promising therapeutic strategy for RA [3]. To this end, local gene delivery can provide an alternative approach to achieve high, long-term expression of biologics, optimizing the therapeutic efficacy and minimizing systemic exposure. Future analogs can be optimized for binding to the human MHC [28]. Our data using adenoX-rCB11-A9 in the CIA animal model convincingly supports the possibility of a collagen-based gene therapy for RA.

## Abbreviations

bp: base pair; CII: type II collagen; CFA: complete Freud's adjuvant; CIA: collagen-induced arthritis; ELISA: enzyme-linked immunosorbent assay; IACUC: Institutional Animal Care and Use Committee; IBC: Institutional Biosafety Committee; IFN: interferon; IL: interleukin; MHC/TCR: major histocompatibility complex/T cell receptor; PCR: polymerase chain reaction; pfu: plaque forming units; PPD: purified protein derivative; RA: rheumatoid arthritis; TNF: tumor necrosis factor.

## Competing interests

The authors declare that they have no competing interests.

## Authors' contributions

BT, DB, and JZ developed the collagen and adenoviral constructs, JAZ and MWG performed the bioimaging studies, HP and KH performed the synovial histology studies, DC, JMS, AHK, and LKM performed the animal studies and participated in the design of the experiments. All authors read and approved the final manuscript.
